# *OsG6PGH1* affects various grain quality traits and participates in the salt stress response of rice

**DOI:** 10.3389/fpls.2024.1436998

**Published:** 2024-07-10

**Authors:** Bo Peng, Yan Liu, Jing Qiu, Jing Peng, Xiaoyu Sun, Xiayu Tian, Zhiguo Zhang, Yaqin Huang, Ruihua Pang, Wei Zhou, Jinhui Zhao, Yanfang Sun, Quanxiu Wang

**Affiliations:** ^1^ College of Life Sciences, Xinyang Normal University, Xinyang, China; ^2^ College of Agronomy, Xinyang Agriculture and Forestry University, Xinyang, China; ^3^ Henan Lingrui Pharmaceutical Company Limited, Xinyang, China; ^4^ School of Pharmacy, Xinyang Agriculture and Forestry University, Xinyang, China

**Keywords:** rice, *OsG6PGH1*, grain quality, proteome, salt stress

## Abstract

Cytoplasmic 6-phosphogluconate dehydrogenase (G6PGH) is a key enzyme in the pentose phosphate pathway that is involved in regulating various biological processes such as material metabolism, and growth and development in plants. However, it was unclear if *OsG6PGH1* affected rice grain quality traits. We perform yeast one-hybrid experiments and reveal that *OsG6PGH1* may interact with *OsAAP6*. Subsequently, yeast *in vivo* point-to-point experiments and local surface plasmon resonance experiments verified that *OsG6PGH1* can bind to *OsAAP6*. *OsG6PGH1* in rice is a constitutive expressed gene that may be localized in the cytoplasm. *OsAAP6* and protein-synthesis metabolism-related genes are significantly upregulated in *OsG6PGH1* overexpressing transgenic positive endosperm, corresponding to a significant increase in the number of protein bodies II, promoting accumulation of related storage proteins, a significant increase in grain protein content (GPC), and improved rice nutritional quality. *OsG6PGH1* positively regulates amylose content, negatively regulates chalkiness rate and taste value, significantly affects grain quality traits such as appearance, cooking, and eating qualities of rice, and is involved in regulating the expression of salt stress related genes, thereby enhancing the salt-stress tolerance of rice. Therefore, *OsG6PGH1* represents an important genetic resource to assist in the design of high-quality and multi-resistant rice varieties.

## Introduction

Rice (*Oryza sativa* L.) is a globally important food crop, upon which more than half of the world’s and more than two-thirds of China’s populations rely as a dietary staple (Wang et al., 2018; Zhao et al., 2020; Li et al., 2022a). With continued improvement in societal living standards, the demand for high-quality rice is increasing (Zhou et al., 2019). Rice grain quality is measured in terms of its physical, chemical, and nutritional characteristics during growth, processing, storage, and consumption. These characteristics affect the nutritional value, taste, and processing performance of rice, and its appearance, nutritional, milling, processing, cooking, and eating qualities (Li et al., 2018; Custodio et al., 2019; Zhou et al., 2020; Zhao et al., 2022a).

Rice appearance is mainly affected by grain size and shape, chalkiness, and translucency. Nutritional quality is mostly affected by protein, starch, fatty and amino acid, vitamin, and mineral contents. The protein in rice is high in quality, and easily digested and absorbed in the gut (Peng et al., 2021; Li et al., 2022b; Gopika et al., 2022; Riaz et al., 2018). Grain protein is the second largest nutritional component after carbohydrates, and its content largely affects nutritional quality. An increase in grain protein content (GPC) improves the nutritional quality of rice. Based on solubility, grain proteins can be divided into glutelins, albumins, prolamins, and globulins (He et al., 2013; Peng et al., 2014; Chen et al., 2018). Milling and processing qualities mainly affect the performance of rice during processing, and are based on percentages of brown, milled, and head rice (Balindong et al., 2018). The brown rice rate directly affects processing quality, and indirectly its edible quality (Tong et al., 2019). The head rice rate is a grading indicator for grain quality. Cooking and eating qualities are the sensory and physicochemical properties of rice during cooking (e.g., extensibility, water absorption, swelling, gelatinization, color, form, aroma, and palatability) (Zhou et al., 2019; Shi et al., 2022), and are measured using amylose content, gelatinization temperature, and gel consistency (Zhou et al., 2019; Bao et al., 2023). Aspects of the cooking and eating quality are of greatest concern to consumers.

Amino acid permease (AAP) belongs to a family of amino acid transporters. *AAPs* play important roles in amino acid transport throughout plant tissues (Tegeder and Hammes et al., 2018). A total of 19 *OsAAPs* have been identified from rice, and most of them have been cloned and functionally identified. Of them, *OsAAP1* and *OsAAP4* regulate absorption and redistribution of neutral amino acids, thereby regulating rice growth, development, and yield (Ji et al., 2020; Fang et al., 2021; Erinaldo et al., 2022); *OsAAP3* and *OsAAP5* mainly transport alkaline amino acids, and have a negative effect on rice tillering and yield (Lu et al., 2018; Wang et al., 2019); *OsAAP6* positively regulates GPC, thereby affecting the nutritional quality of rice (Peng et al., 2014); *OsAAP7* and *OsAAP16* can transport most amino acids (excepting aspartic acid and β-alanine) (Taylor et al., 2015); *OsAAP8* mutation significantly increases GPC and amino acid contents (Peng et al., 2024); *OsAAP10* and *OsAAP11* regulate starch and protein contents in rice grains by affecting the transport of acidic and neutral amino acids, respectively (Wang et al., 2020; Yang et al., 2023a); *OsAAP13* is mainly involved in amino acid transportation in above-ground parts of rice, and plays an important role in rice growth, development, and stress regulation (Nie et al., 2023); *OsAAP14* is involved in nitrogen metabolism and positively regulates rice axillary bud growth, tillering, and yield (Yang et al., 2022); *OsAAP15* affects rice yield by regulating panicle branch development (Yang et al., 2023b); and *OsAAP17*-related alleles positively regulate yield per plant in *indica* rice (Itishree et al., 2023). Thus, *OsAAPs* play important roles in regulating rice yield and quality traits.

The pentose phosphate pathway (PPP) is important for sugar metabolism in plants; its main physiological function is to produce reduced NADPH and generate pentose phosphate to participate in nucleic acid metabolism. PPP is the main source of NADPH in eukaryotic cells, and is involved in regulating metabolic intermediates in the biosynthesis process, which can participate in the synthesis of substances such as fatty and amino acids (Wang et al., 2017; Li et al., 2022c). 6-phosphogluconate dehydrogenase (G6PGH/6PGDH) is a key enzyme in the PPP, catalyzing the production of 5-phosphoribose and NADPH from 6-phosphogluconate. *G6PGH* has been successfully isolated and cloned from plants such as soybeans, corn, tomatoes, apples, *Arabidopsis*, rice, and wheat (Hanks et al., 1983; Tanksley and Kuehn, 1985; Bailey-Serres and Nguyen, 1992; Huang et al., 2003; Hölscher et al., 2016; Su et al., 2016). *G6PGH* participates in the plant’s response to various abiotic stresses, and plays an important role in responses to temperature extremes, salt, heavy metals, and osmotic and other stresses. In maize, *6PGDH* can significantly increase yield under high temperature stress. After a double mutation of *6pgd1* and *6pgd2* in corn, the homozygous mutant showed delayed growth (Bailey-Serres and Nguyen, 1992); after the *PGD3* mutation, the mutant exhibited severe granule filling and defective embryonic phenotype (Ribeiro et al., 2020). Overexpression of *6PGDH* in *Arabidopsis* significantly enhances cold tolerance (Tian et al., 2021), and can accelerate growth of callus tissue in apples (Zhu et al., 2019). Transcription levels of two types of *OsG6PGH* isolated and cloned in rice (cytoplasmic 6-phosphogluconate dehydrogenase (*OsG6PGH1*) and plastid 6-phosphogluconate dehydrogenase (*OsG6PGH2*)) were significantly upregulated in rice seedlings under drought, high salt, cold, and abscisic acid treatment, indicating that *OsG6PGH* plays an important role in the response of rice to abiotic stress (Huang et al., 2003; Hou et al., 2007). However, it remained unclear if *OsG6PGH* was involved in biological processes such as rice growth, development, and substance metabolism, or if it affected grain quality.

We identified a major quantitative trait locus gene *OsAAP6* that positively regulated GPC and affected rice nutritional quality (Peng et al., 2014, 2018). To gain a deeper understanding of the molecular mechanism by which *OsAAP6* regulated GPC, we determined *OsG6PGH1* to possibly interact with it (Peng et al., 2023). We further validated the ability of *OsG6PGH1* to bind to *OsAAP6* through *in vivo* point-to-point experiments in yeast and localized surface plasmon resonance experiments. In overexpressing *OsG6PGH1* plants, we report *OsG6PGH1* as capable of regulating the expression of *OsAAP6* to affect the contents of the main storage substances in rice grains, and to have important effects on grain quality traits. Simultaneously, *OsG6PGH1* participates in biological processes such as the salt-stress response. These results are important for molecular design and breeding of high-quality and multi-resistant rice varieties.

## Materials and methods

### Point-to-point validation in yeast cells

We first simultaneously transformed the plasmid pGADT7-Rec2-*OsG6PGH1* carrying the target gene *OsG6PGH1* and the bait plasmid pHIS2-*OsAAP6* into yeast receptive cell Y187. Transformed yeast cells were cultured on SD-Trp-Leu solid medium at 30°C for 3–5 d (Peng et al., 2023). Select yeast monoclonal antibodies from this medium were inoculated into SD-Trp-Leu liquid medium. Cultivation was continued at 30°C for 12 h. The OD600 value of the yeast solution was adjusted for all samples to 0.4 by adding ddH_2_O to ensure consistency in experimental conditions. Next, the standardized yeast solution onto SD-Leu-Trp-His+150 mmol·L^-1^ 3AT medium, and cultured at 30°C for 3–4 d to further validate the interaction between yeast clones and bait proteins (Peng et al., 2023; Zhang et al., 2024).

### Localized surface plasmon resonance

Binding analysis of *OsG6PGH1* and *OsAAP6* was performed using the OpenSPR surface plasmon resonance biosensor (Nicoya Life Science Inc., Kitchener, Canada). Using a standard coupling reaction of 1-ethyl-3-(3-dimethylpropyl)-carbodiimide (EDC) and N-hydroxysuccinimide (NHS), the *OsAAP6* was fixed at a concentration of 50 μg·ml^-1^ onto the activated COOH sensor chip (Peng et al., 2023). A blocking solution was then injected into the sensor chip to reduce the possibility of non-specific binding. Then, prokaryotic total cell lysate protein solutions at concentrations of 15, 22.5, and 30 μg·ml^-1^ were introduced to the sensor chip. As the sample flows through the chip, *OsAAP6* contained in it binds specifically to previously fixed antibodies, and is captured by the sensor (Liu et al., 2019). To detect the interaction between *OsAAP6* and *OsG6PGH1*, we injected 50 μg·ml^-1^ IgG as a negative control and *OsG6PGH1* antibody as a target protein into the sensor chip. By comparing the response signals of IgG (non-specific binding) and *OsG6PGH1* antibody (with potential specific binding), it can be determined if *OsG6PGH1* has interacted with *OsAAP6*.

### Creation of *OsG6PGH1* transgenic rice lines

To create mutants targeting the deletion of *OsG6PGH1* based on CRISPR/Cas9 gene-editing tool, we commissioned Wuhan Astronomical Biotechnology Co., Ltd. to conduct genetic transformation experiments. PCR and Sanger sequencing techniques were used to detect and analyze the mutant sequence (Peng et al., 2024), ensuring the successful knockout of *OsG6PGH1*.

The cDNA fragment of *OsG6PGH1* was cloned onto the intermediate vector PMD-18T to obtain the PMD-18T-*OsG6PGH1* recombinant plasmid. Sequencing validation was then performed on the recombinant plasmid to ensure the accuracy of the *OsG6PGH1* sequence. After verifying the accuracy, the target fragment was cut from PMD-18T-*OsG6PGH1* and connected to the pCMBIA1301s overexpression vector to construct the overexpression plasmid pCMBIA1301s-*OsG6PGH1*. Wuhan Astronomical Biotechnology Co., Ltd. was again entrusted to use *Agrobacterium-*mediated genetic transformation to introduce this overexpression plasmid into rice cells, to overexpress the *OsG6PGH1*.

### RNA extraction and real-time quantitative polymerase chain reaction

Total RNA was extracted using TRIzol reagent according to manufacturer instructions (TAKARA) (Peng et al., 2024). A reverse transcription kit (R202-02 HyperScript III RT SuperMix for qPCR with gDNA Remover) was used to reverse transcribe RNA into cDNA. qRT-PCR was performed using 2 SYBR Green Fast qPCR Master Mix (YiFeiXue Bio Technology) on quantitative analyzer (ABI7300), with a reaction program involving a predenaturation step at 95°C for 30 s, and an amplification reaction of 40 cycles, each including 95°C denaturation for 15 s, and a 60°C annealing extension for 30 s. To accurately evaluate changes in expression of target genes, *β-actin* was used as an internal reference gene, and relative expression levels were standardized using the 2*^−ΔΔCT^* calculation method (Wang et al., 2022). Three biological replicates were performed for each reaction using primers detailed in **Supplementary Tables 1–4**.

### Expression patterns

Based on the cDNA sequence of *OsG6PGH1*, we designed and detected primers using the NCBI website. Total RNA was extracted from root, stem, leaf, sword leaf, glume, stem node, and endosperm tissues, and reverse transcribed into cDNA. Expression levels of *OsG6PGH1* in tissues were detected by qRT-PCR (Li et al., 2023). *β-Actin* was used as an internal reference gene in each replicated experiment.

### Subcellular localization

Protoplasts were first extracted from rice seedlings cultured in darkness for 14 d. The subcellular localization vector *OsG6PGH1*-*GFP* and plasma membrane (*PAT2*-*RFP*) and nuclear (*Ghd7*-*RFP*) markers were co-transformed into the protoplasts of rice seedlings. Fluorescence signals in protoplasts were detected using confocal fluorescence microscopy (Peng et al., 2023); all fluorescence experiments were independently observed at least three times.

### Plant growth status and trait determination

Field experiments were performed on rice plants at Xinyang Normal University, Henan Province. Planting density was 16.5 × 26.0 cm, with the field managed in accordance with local agricultural standards. Before measurement, harvested grains were air dried, and then stored at room temperature for at least 3 months. Fully grouted grains were used to detect rice quality and yield traits (Li et al., 2014). A near-infrared grain analyzer (INFRAEC Nova) was used to detect protein, amylose, and free fatty acid contents, and taste value (Peng et al., 2023). The gelatinization temperature of grain was measured using the alkaline digestion method (Peng et al., 2024), and polished rice gel consistency was determined following People’s Republic of China (GB/T17891-1999, high-quality rice) national standards.

### Transmission electron microscopy

The endosperm of 10 DAF rice was fixed in 2.5% glutaraldehyde with 0.2 mol L^−1^ phosphate buffer (pH 7.2) for at least 12 h. Fixed endosperm tissue was cut into 1 mm sections using an ultra-thin slicer (Power Tome XL, RMC). Sections were examined by TEM (H-7650, Hitachi). ImageJ software (NIH) was used to calculate the quantity and area of PBs in each TEM sample image (Peng et al., 2014).

### Observation by optical and scanning electron microscopy

The middle part of the grain was gently tapped with the back of a knife to allow natural breakage; a sharp blade was then used to cut the broken part to form a thin sheet of ~2–3 mm thickness. Slices were then divided into two: one part to observe the chalky area beneath a regular optical microscope; the other to be fixed on a copper sample stage with conductive adhesive, sprayed with platinum using a HUS-5GB vacuum coating machine, and examined by scanning electron microscope (Hitachi S-4800) (Xu et al., 2022).

### Proteomic analysis

Both *OsG6PGH1*(OX) positive and negative plants grew in the same environment. Endosperms were simultaneously collected, and then ground for proteomic sequencing (replicated three times per sample). Total protein was first extracted, and its concentration, purity, and integrity were examined to ensure sample usability. Obtained data were filtered to obtain clean readings aligned with the reference proteome sequence. Statistical tests were performed to obtain DEPs based on the annotation file of the proteome and reading position (i.e., expression level) compared with the proteome. Finally, Clusters of Orthologous Genes functional annotation, Gene Ontology, Kyoto Encyclopedia of Genes and Genomes, and Pfam enrichment analyses were performed on DEPs to identify biological processes and metabolic pathways of protein enrichment (Xiong et al., 2019).

### Salt stress test

After peeling and disinfecting *OsG6PGH1* transgenic seeds, they were inoculated into 1/2 MS solid culture medium. After 7 d, rice seedlings with consistent growth were selected and placed into a hydroponic medium for acclimation for 7 d. Seedlings cultured for 14 d were inoculated in 50 mmol L^−1^ and 100 mmol L^−1^ concentrations of NaCl medium, and placed into a 28°C constant temperature light incubator. Nutrient solutions were changed every 2 d to prevent bacterial contamination (Zhao et al., 2022b). Seedling growth was monitored daily.

## Results

### *OsG6PGH1* can bind to *OsAAP6*

**Figure 1:**
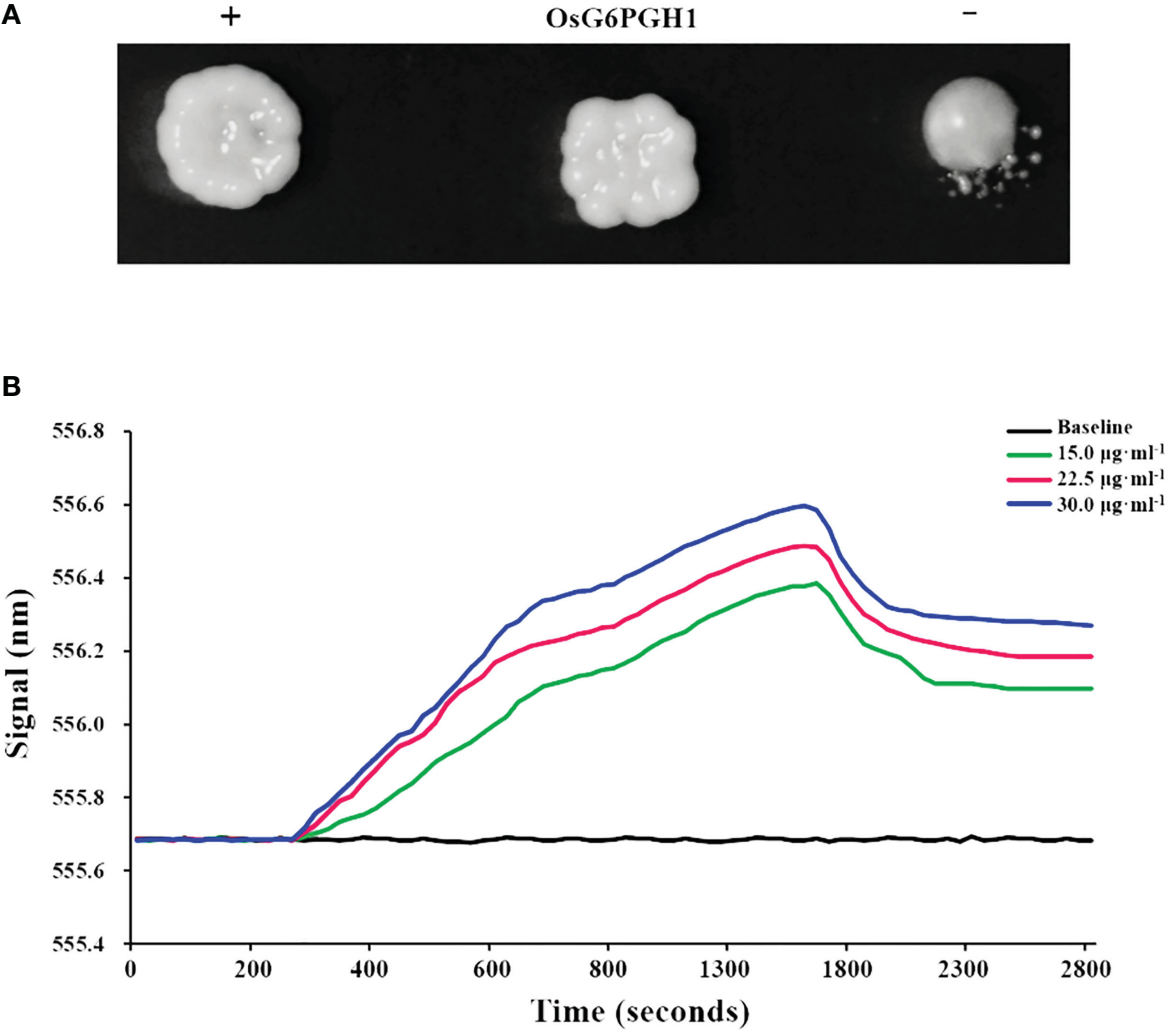
Verification of interactions between *OsG6PGH1* and OsAAP6. **(A)** Yeast *in vivo* point-to-point experiment: +, positive control; −, negative control. **(B)** *In vitro* validation using localized surface plasmon resonance experiments.

A total of 92 regulatory factors that may interact with *OsAAP6* were screened through yeast one- hybrid experiments (Peng et al., 2023). One regulatory factor, the plasmid pGADT7-Rec2-*G6PGH1*, along with the bait plasmid pHIS2-*OsAAP6*, were transferred into yeast receptive cell Y187. On solid culture media, with the addition of 3AT inhibitors, the *OsG6PGH1* yeast colony and positive control yeast colony (+) both grew well, but there were significant differences between them and the negative control (−) (**Figure 1A**). This indicates that *OsG6PGH1* can interact with OsAAP6 in yeast.

To verify the interaction between *OsG6PGH1* and *OsAAP6*, prokaryotic expression vectors pET-28a-*OsG6PGH1* and pET-28a were transfected into *Escherichia coli* competent cells BL21 (DE3). In local surface plasmon resonance experiments, the curves for three concentrations all significantly trended upward at 280 s. This change indicated that an interaction occurred between *OsG6PGH1* and *OsAAP6*, forming a complex (*OsG6PGH1*–*OsAAP6*). At 1650 s, there was a significant downward trend in curves for each concentration, indicating that the *OsG6PGH1*–*OsAAP6* complex had undergone dissociation (**Figure 1B**). These dynamic changes indicate that *OsG6PGH1* can bind to *OsAAP6 in vitro*.

### *OsG6PGH1* is a constitutive expression gene and the encoded protein may be localized in the cytoplasm

**Figure 2:**
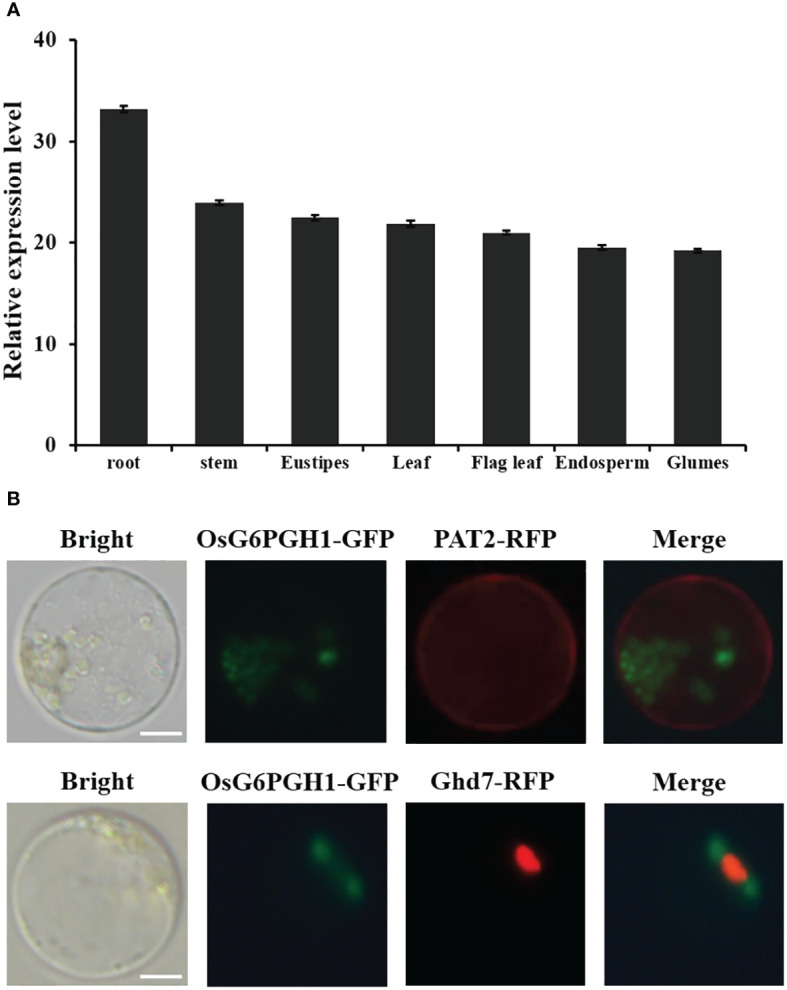
Analysis of *OsG6PGH1* expression patterns and subcellular localization in rice. **(A)** Detection and analysis of expression levels in different tissues. **(B)** *OsG6PGH1*-GFP is not co-localized with either the membrane marker protein PAT2-RFP or nuclear marker protein Ghd7-RFP. Scale bars, 5 μm; error bars, standard error of the mean (SEM).

To investigate expression levels of *OsG6PGH1* in different rice tissues, RNA was extracted from root, stem, stem node, leaf, sword leaf, endosperm, and glume tissues. Expression patterns were analyzed using qRT-PCR. *OsG6PGH1* was expressed in each tissue type (**Figure 2A**), and this result was in accordance with the distinct tissue expressions of *OsG6PGH1* (LOC_Os06g02144) on CREP (**Supplementary Figure 1**), indicating it is a constitutive expression gene. Expression levels of *OsG6PGH1* were higher in roots, and lower in the endosperm and glume (**Figure 2A**).

To investigate localization of *OsG6PGH1* in cells, *OsG6PGH1* was fused with the green fluorescent protein gene (*GFP*) (*OsG6PGH1-GFP*) and simultaneously co-expressed with the cell-membrane-localization marker gene *PAT2*-RFP and nuclear marker gene *Ghd7*-RFP in rice protoplasts, and observed using laser confocal electron microscopy. *OsG6PGH1*-GFP was not co-localized with membrane marker protein PAT2-RFP or nuclear marker protein Ghd7-RFP (**Figure 2B**), indicating that *OsG6PGH1* was not localized in either the cell membrane or nucleus, but that it may be localized in the cytoplasm.

### Creation of *OsG6PGH1* transgenic rice lines

To investigate the biological function of *OsG6PGH1* in rice, we created CRISPR/Cas9 knockout and overexpressing transgenic lines of *OsG6PGH1*. Based on the wild-type Zhonghua 11 (ZH11), a target site for *OsG6PGH1* was designed. CRISPR/Cas9 gene editing tool was used to target and induce the fusion of sgRNA and Cas9 protein, and specific splicing was performed on the two PAM sites of *OsG6PGH1*. A gene edited expression vector was constructed, and 20 CRISPR/Cas9 gene edited plants were obtained through *Agrobacterium-*mediated genetic transformation (including 16 *OsG6PGH1* positive mutants) (**Supplementary Figure 2A**). DNA was extracted from the leaves of the *OsG6PGH1* mutants, and PCR amplification and DNA sequencing were performed; two types of mutations were found in the cDNA target sequence of *OsG6PGH1*: *Osg6pgh1-1* (+1 bp) and *Osg6pgh1-2* (+2 bp) (**Supplementary Figure 2B**). The target sequence of *Osg6pgh1-1* has a 1 bp insertion and 3 bp substitution in its second exon, resulting in a frameshift mutation, and *Osg6pgh1-2* has a 2 bp insertion and 4 bp substitution in its first and second exons, which also causes a frameshift mutation.

To obtain transgenic plants with *OsG6PGH1* overexpression, we connected the cDNA of *OsG6PGH1* to the intermediate vector PMD-18T to obtain PMD-18T-*OsG6PGH1*. After sequencing verification, the target fragment was connected to the pCMBIA1301s overexpression vector to obtain the *OsG6PGH1* overexpression plasmid pCMBIA1301s-*OsG6PGH1*. Through *Agrobacterium-* mediated genetic transformation, 20 transgenic plants with *OsG6PGH1* overexpression (*OsG6PGH1*(OX)) were obtained (including 15 positive plants) (**Supplementary Figure 2C**).

### Overexpression of *OsG6PGH1* affects the content of major nutrients in rice

**Figure 3:**
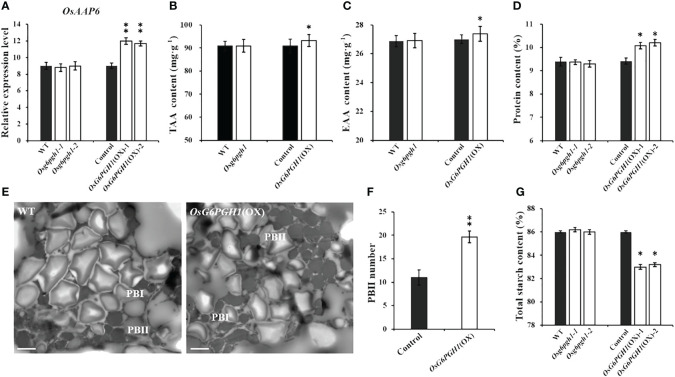
Detection and analysis of major nutrients in transgenic rice grains with *OsG6PGH1*. **(A)** Detection and analysis of expression levels of *OsAAP6* in *OsG6PGH1* transgenic endosperm. *OsG6PGH1* transgenic plant: **(B)** total amino acids, **(C)** essential amino acids, and **(D)** grain protein content. **(E)** transmission electron microscopy image of endosperm of *OsG6PGH1*(OX) 10 days after fertilization; and analysis of **(F)** protein body II, and **(G)** total starch content. Significant differences are based on two tailed *t*-tests, where **P ≤ 0.01, *P ≤ 0.05. WT, Wild type; Control, *OsG6PGH1*(OX) negative control. Scale bars, 2 μm; error bars, standard error of the mean (SEM), n ≥ 50.

We performed qRT-PCR analysis on expression levels of *OsAAP6* in the endosperm of *OsG6PGH1* mutants (M_2_) and overexpressing transgenic F_2_ lines. There was no significant change in expression levels of *OsAAP6* in the endosperm of *OsG6PGH1* homozygous mutants, while expression levels significantly increased in the *OsG6PGH1* overexpressing positive endosperm (**Figure 3A**).

Because of the influence of *OsAAP6* on the distribution of amino acids in grains and its positive regulation of grain protein content, we detected and analyzed amino acid contents and GPC in *OsG6PGH1* mutants (M_2_) and overexpressing transgenic F_2_ grains. *OsG6PGH1* affected the content of some amino acids (e.g., alanine, glutamic acid, leucine, lysine, methionine) (**Supplementary Figure 3A**). Total and essential amino acid contents in grains of *OsG6PGH1*(OX) positive plants significantly increased, while there was no difference in grains of *OsG6PGH1* homozygous mutants (**Figures 3B, C**). Additionally, the GPC in *OsG6PGH1*(OX) positive plants increased significantly, while there was no significant difference in *OsG6PGH1* mutants (**Figure 3D**). To further investigate if *OsG6PGH1*(OX) affected the protein bodies in rice endosperm, we observed the endosperm of *OsG6PGH1*(OX) 10 d after fertilization (DAF) using transmission electron microscopy (TEM). Protein bodies I (PB I) and protein bodies II (PB II) were clearly visible under TEM: PB I had relatively regular circular or elliptical shapes, and PB II had irregular shapes and stained uniformly (**Figure 3E**). Average cross-sectional areas of PB I and II in *OsG6PGH1*(OX) positive endosperms did not differ significantly from those in transgenic negative endosperms, while numbers of PB II significantly increased (**Figure 3F**; **Supplementary Figures 3B–D**). This indicates that overexpression of the *OsG6PGH1* significantly increased the number of PB II in rice endosperm, leading to increased GPC.

To further investigate if *OsG6PGH1* affected the accumulation of nutrients such as starch and free fatty acids in rice, starch and free fatty acid content was measured in *OsG6PGH1* mutants (M_2_) and overexpressing transgenic F_2_ plants. Total starch contents in *OsG6PGH1*(OX) positive grains were significantly reduced, while total starch contents in *OsG6PGH1* homozygous mutants did not change significantly (**Figure 3G**). There was no significant change in free fatty acid content in homozygous mutants of *OsG6PGH1* or *OsG6PGH1*(OX) positive grains (**Supplementary Figure 3E**). Therefore, overexpression of *OsG6PGH1* helps to increase GPC, but not the accumulation of starch in grains.

### *OsG6PGH1* participates in regulating expression of genes related to protein and starch metabolism in grains

**Figure 4:**
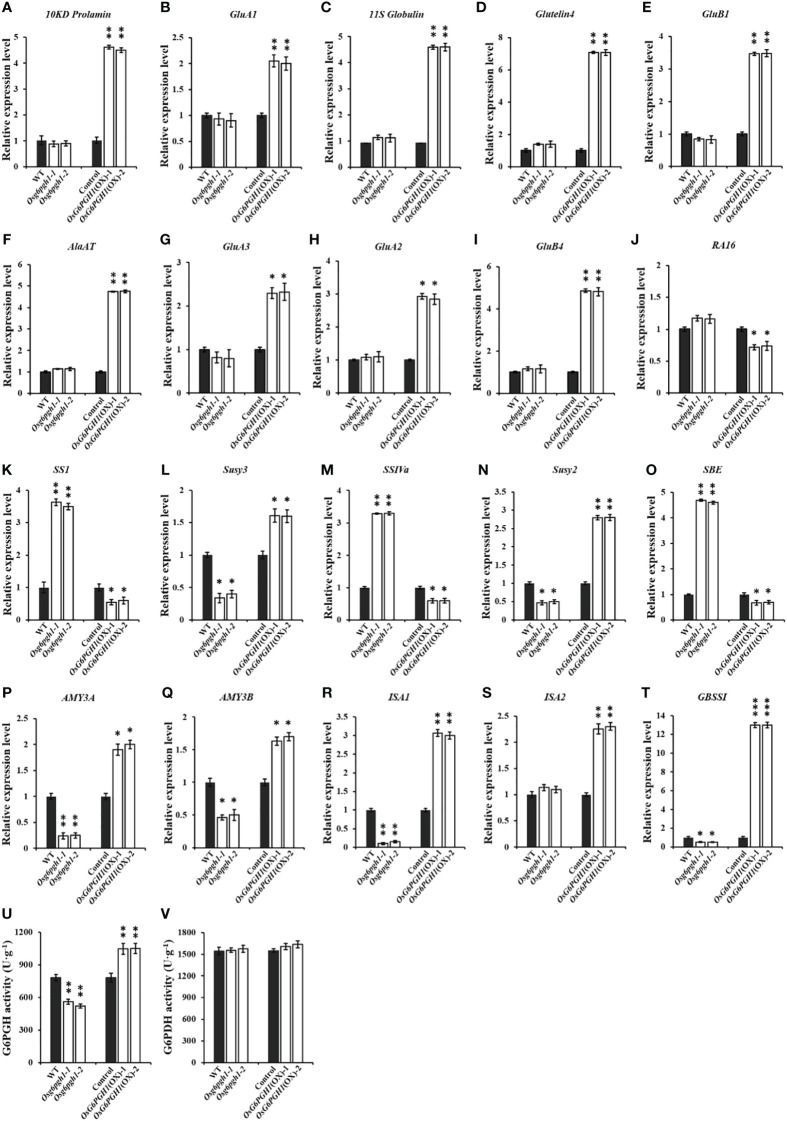
Expression and enzyme activities of related genes in *OsG6PGH1* transgenic rice endosperm. **(A–J)** qRT-PCR analysis of protein-metabolism-related genes; **(K–T)** qRT-PCR analysis of starch-metabolism-related genes; and detection and analysis of enzyme activity of endosperm **(U)** 6PGDH and **(V)** G6PDH in *OsG6PGH1* transgenic rice lines 10 DAF. Significant differences are based on two tailed *t*-tests, where ***P ≤ 0.001, **P ≤ 0.01, *P ≤ 0.05. WT, Wild type; Control, *OsG6PGH1*(OX) negative control. Error bars, standard error of the mean (SEM).

Because of the significant impact of *OsG6PGH1*(OX) on accumulation of protein and starch in grain, we examined expression levels of protein-and starch-metabolism-related genes in *OsG6PGH1* mutants and overexpressed transgenic F_2_ endosperm. Gene expression levels correlated positively with grain protein synthesis and accumulation metabolism, with *10KD Prolamine*, *GluA1*, *11S Globulin*, *Glutelin 4*, *GluB1*, *AlaAT*, *GluA3*, *GluA2*, *GluB4* significantly upregulated in the positive endosperm of *OsG6PGH1*(OX), but not in *OsG6PGH1* homozygous mutants (**Figures 4A–J**). Expression levels of starch-synthesis-related genes (*Susy2*, *Susy3*) and starch-degradation metabolism-related genes (*AMY3A*, *AMY3B*, *ISA1*) were significantly downregulated in *OsG6PGH1* homozygous mutants, and significantly upregulated in *OsG6PGH1*(OX) positive plants. Expression levels of genes related to starch synthesis (*SS1*, *SSIVa*, *SBE*) were significantly upregulated in *OsG6PGH1* homozygous mutants, while expression levels were the opposite in *OsG6PGH1*(OX) positive plants (**Figures 4K–S**). The granule-bound starch synthase gene (*GBSSI*) related to amylose synthesis was significantly downregulated in *OsG6PGH1* homozygous mutants and significantly upregulated in *OsG6PGH1*(OX) positive plants (**Figure 4T**). These results are consistent with a phenotype with significant increases in GPC and a significant decrease in starch content in rice grains with *OsG6PGH1*(OX).

To investigate the effect of changes in *OsG6PGH1* on the activity of upstream and downstream enzymes in the PPP, we measured the activity of 6-phosphogluconate dehydrogenase (G6PGH) and its upstream rate-limiting enzyme glucose-6-phosphate dehydrogenase (G6PDH) in the endosperm of rice 10 DAF. Activity of G6PGH in the endosperms of *OsG6PGH1* transgenic F_2_ plants changed significantly: the activity of G6PGH in endosperm of *OsG6PGH1* homozygous mutants was significantly reduced, and significantly enhanced in *OsG6PGH1*(OX) positive endosperms (**Figure 4U**); the activity of the upstream rate-limiting enzyme G6PDH did not change significantly in *OsG6PGH1* transgenic plants (**Figure 4V**).

### *OsG6PGH1* affects the cooking and processing quality of rice

**Figure 5:**
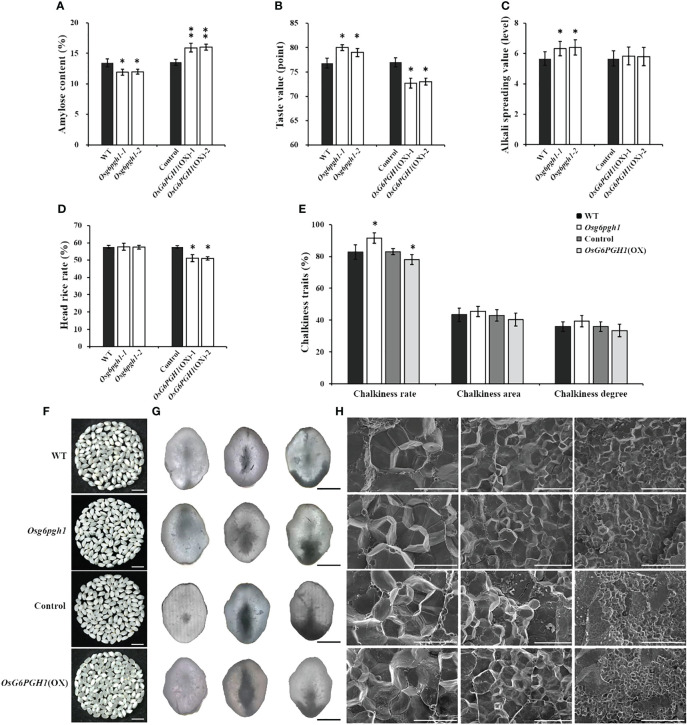
Analysis of cooking and eating, processing, and appearance quality traits of *OsG6PGH1* transgenic rice. Detection and analysis of: **(A)** amylose content, **(B)** taste value, **(C)** gelatinization temperature; **(D)** head rice rate, **(E)** chalkiness traits, and **(F)** endosperm chalkiness characteristics. Scale bars, 1 cm. Images of: **(G)** chalkiness under optical microscope; columns from left (transparent), central (heart white), and right (belly white); scale bars, 1 mm; **(H)** Scanning electron microscopy of endosperm; scale bars, left to right: 1 mm, 10 μm, 20 μm, and 100 μm. Significant differences are based on two-tailed *t*-tests, where **P ≤ 0.01, *P ≤ 0.05. WT, Wild type; Control, *OsG6PGH1* overexpression transgenic negative control. Error bars, standard error of the mean (SEM).

To investigate if *OsG6PGH1* affects the cooking and eating quality of rice, we tested amylose content, taste value, gel consistency, and gelatinization temperature of *OsG6PGH1* mutants and overexpressing transgenic F_2_ plants. Compared with wild type rice, the amylose content of *OsG6PGH1* homozygous mutants was significantly lower, and taste value and gelatinization temperature were significantly higher. Amylose contents of *OsG6PGH1*(OX) positive grains were significantly higher, the taste value was significantly lower, and the gelatinization temperature did not change significantly (**Figures 5A–C**). Gel consistency of *OsG6PGH1* mutants and overexpressing transgenic rice showed no significant changes (**Supplementary Figure 4A**).

Because amylose contents of *OsG6PGH1* mutants and overexpressing transgenic rice changed significantly, we analyzed their milling quality traits. The head rice rate of *OsG6PGH1*(OX) positive F_2_ rice significantly decreased (**Figure 5D**), while the brown and milled rice rates showed no significant change (**Supplementary Figures 4B, C**). Compared with wild type rice, there was no significant change in milling quality traits of *OsG6PGH1* homozygous mutant rice (**Figure 5D**; **Supplementary Figures 4B, C**). These results indicate that upregulation of *OsG6PGH1* expression does not improve rice cooking or eating quality, decreases the head rice rate, and affects the grain processing quality.

### *OsG6PGH1* negatively regulates rice chalkiness rate

Chalkiness rates of *OsG6PGH1* homozygous mutants increased significantly, while the chalkiness rate of *OsG6PGH1*(OX) rice was significantly reduced (**Figures 5E, F**). There were no significant changes in chalkiness area or degree in *OsG6PGH1* mutants or overexpressing transgenic rice. The morphology and arrangement of starch granules in the endosperm of *OsG6PGH1* mutants and overexpressing transgenic positive rice showed no significant change; both presented a densely arranged polygonal structure (**Figures 5G, H**). Therefore, *OsG6PGH1* negatively regulated the chalkiness rate of grain, but did not affect chalkiness area or degree.

### *OsG6PGH1* does not affect rice yield per plant

To investigate if *OsG6PGH1* affected rice yield, we tested yield-related traits of *OsG6PGH1* mutants (M_2_) and their overexpressing transgenic F_2_ plants. *OsG6PGH1* homozygous mutant plant height was significantly reduced (**Supplementary Figures 5A, B**); there were no significant changes in the number of tillers per plant, number of spikelets per panicle, seed setting rate, thousand grain weight, or yield per plant (**Supplementary Figures 5C–G**). Numbers of spikelets per panicle of *OsG6PGH1*(OX) positive plants significantly decreased (**Supplementary Figure 5D**), and the setting rate significantly increased (**Supplementary Figure 5E**); other yield-related traits showed no significant changes, leading to no overall significant change in rice yield per plant (**Supplementary Figure 5G**). Because *OsG6PGH1*(OX) significantly affected grain protein and starch contents, we examined the grain type of *OsG6PGH1* mutants and overexpressed transgenic seeds. There were no significant changes in grain length, width, or thickness of *OsG6PGH1* homozygous mutants and overexpressing transgenic positive grains (**Supplementary Figure 6**). Therefore, although *OsG6PGH1* affected plant height, number of spikelets per panicle, and seed setting rate, it did not affect rice yield per plant.

### Proteomic analysis of *OsG6PGH1*(OX) transgenic endosperms

The GPC in *OsG6PGH1*(OX) positive grains significantly increased. To evaluate the accumulation of different storage proteins in grains, we performed proteomic analysis on *OsG6PGH1*(OX) endosperm. Upregulated proteins were screened using two methods: differential multiple (|log2 (Fold change)| ≥ 1.50) and significance level (*P*-value ≤ 0.05). Downregulated proteins were screened through two methods: differential multiple (|log2 (Fold change)| ≤ 1/1.50) and significance level (*P*-value ≤ 0.05). A total of 156 differentially expressed proteins (DEPs) were screened (**Supplementary Figure 7A**).

To understand the structure and function of these DEPs, functional annotation was performed using the COG database. Functions of DEPs were mainly related to amino acid transport and metabolism, carbohydrate transport and metabolism, and protein post-translational modification (**Supplementary Figure 7B**). GO functional enrichment analysis of these DEPs revealed significant differential enrichment in *OsG6PGH1*(OX) positive endosperms to occur mainly in cellular components and organelles, participation in metabolic processes, cellular processes, and other biological processes, with molecular functions such as binding and catalytic activity (**Supplementary Figure 7C**). KEGG pathway analysis on these DEPs revealed carbon metabolism, glycolysis/gluconeogenesis, starch and sucrose metabolism, and carbon fixation by photosynthetic organisms, to be pathways where DEPs were more concentrated in *OsG6PGH1*(OX) positive endosperms (**Supplementary Figure 7D**). Domain annotation and enrichment were performed on these DEPs through the Pfam database; protease inhibitors, seed storage, and LTP family were mostly enriched in *OsG6PGH1*(OX) positive endosperms (**Supplementary Figure 7E**). Results of proteomic analysis are consistent with upregulation of protein- and amylose-synthesis-related genes, and downregulation of amylopectin-metabolism-related genes in the positive endosperm of *OsG6PGH1*(OX)(**Figure 4**; **Supplementary Table 5**). This suggests that overexpression of *OsG6PGH1* may promote protein synthesis and accumulation in the endosperm, while inhibiting corresponding starch synthesis and metabolic pathways.

### *OsG6PGH1* enhances rice tolerance to salt stress

**Figure 6:**
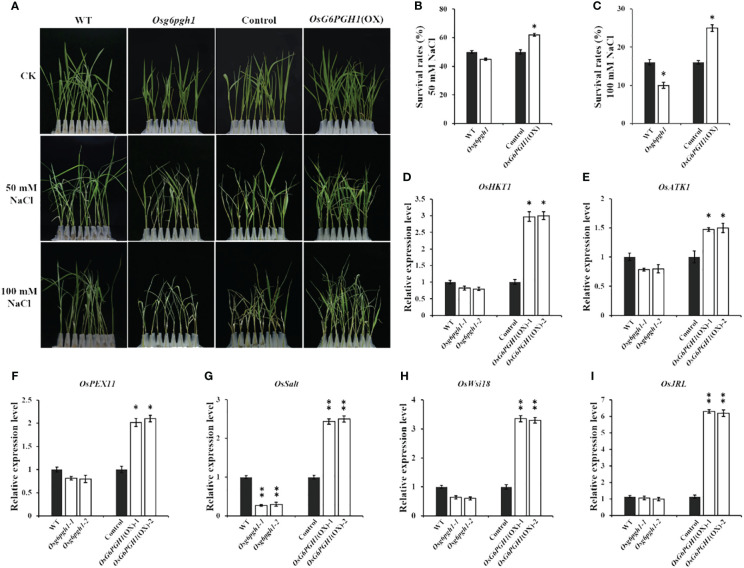
Salt stress tests and expression analysis of related genes in *OsG6PGH1* transgenic rice seedlings. **(A)** Salt stress test. Survival rate of rice seedlings at **(B)** 50 mmol·L^−1^ NaCl, and **(C)** 100 mmol·L^−1^ NaCl. **(D–I)** qRT-PCR detection and analysis of salt stress-related genes. Significant differences are based on two-tailed *t*-tests, where **P ≤ 0.01, *P ≤ 0.05. WT, Wild type; Control, *OsG6PGH1* overexpression transgenic negative control. Error bars, standard error of the mean (SEM).

Because *OsG6PGH1*, as a key gene in the PPP, may be involved in the salt stress response of rice, we performed salt stress treatment experiments on *OsG6PGH1* mutants (M_2_) and *OsG6PGH1*(OX) transgenic F_2_ seedlings. Rice seedlings grown for 14 d in culture media were exposed to concentrations of 50 mmol·L^−1^ and 100 mmol·L^−1^ NaCl for 7 d. The survival rate of *OsG6PGH1* homozygous mutant seedlings was significantly reduced compared with wild-type rice at 100 mmol·L^−1^ NaCl, but that of *OsG6PGH1*(OX) positive seedlings increased significantly in both salt stress treatments. This indicates that *OsG6PGH1* improves salt stress resistance of rice seedlings (**Figures 6A–C**).

To further explore the molecular mechanism of *OsG6PGH1* regulating salt stress resistance in rice seedlings, we examined expression levels of six salt stress-related genes (*OsPEX11*, *OsSalt*, *OsWsi18*, *OsJRL*, *OsHKT1*, *OsATK1*) in *OsG6PGH1* mutants and *OsG6PGH1*(OX) seedlings. qRT-PCR results revealed expression levels of these genes to be significantly increased in *OsG6PGH1*(OX) positive seedlings, while *OsSalt* was significantly reduced in *OsG6PGH1* homozygous mutant plants (**Figures 6D–I**). These results are consistent with observations that salt stress resistance of *OsG6PGH1* homozygous mutant seedlings is significantly reduced, while the salt stress resistance of transgenic positive rice seedlings is significantly enhanced.

## Discussion

*AAPs* are involved in various physiological metabolic processes in plants, and affect rice growth and development (Fang et al., 2021). In the rice genome, 19 *OsAAPs* (*OsAAP1*–*19*) have been identified, with most of them isolated, cloned, and identified (Peng et al., 2014; Taylor et al., 2015; Lu et al., 2018; Wang et al., 2019; Ji et al., 2020; Wang et al., 2020; Fang et al., 2021; Erinaldo et al., 2022; Yang et al., 2022; Yang et al., 2023a, b; Peng et al., 2024). Among them, *OsAAP6* is a major quantitative trait locus gene that positively regulates GPC and nutritional quality of rice (Peng et al., 2014, 2019). To further elucidate the regulatory mechanism of *OsAAP6*, a total of 92 potential regulatory factors that may interact with it were screened through yeast one-hybrid experiments (Peng et al., 2023). The ability of *OsG6PGH1* to bind to *OsAAP6* was then validated through yeast *in vivo* point-to-point rotation experiments and local surface plasmon resonance experiments (**Figure 1**); it was involved in regulating expression of *OsAAP6* in *OsG6PGH1*(OX). These results provide key clues for the in-depth analysis of *OsAAP6* regulation of GPC, but also open up new possibilities for using *OsAAP6* as a gene resource in molecular design breeding.

*G6PGH* is a key gene in the PPP, with multiple biological functions. After the double mutation of *6pgd1* and *6pgd2* in corn, the growth of homozygous individuals with the mutation was delayed (Bailey-Serres and Nguyen, 1992); overexpression of *G6PGH* in apples can accelerate growth of callus tissue (Zhu et al., 2019). We obtained *OsG6PGH1* mutants using CRISPR/Cas9 gene-editing tool and created *OsG6PGH1*(OX) transgenic plants. *OsG6PGH1* homozygous mutant plant height was significantly reduced, as were the number of spikelets per panicle in *OsG6PGH1*(OX) transgenic positive plants, while the seed setting rate increased significantly. Notably, there was no significant change in rice yield per plant in *OsG6PGH1* homozygous mutants or overexpressing transgenic positive rice. Therefore. *OsG6PGH1* may be involved in regulating rice growth and development, but not yield per plant.

Rice grains are rich in amino acids, with a relatively balanced composition (20 types of amino acids, including 8 essential amino acids) (Yang et al., 2022). Grain protein is also high-quality, and easily digestible and absorbed (Peng et al., 2023). GPC is a key factor in the nutritional quality of rice (Xia et al., 2022). Therefore, exploring genes related to GPC and analyzing their functions is important for molecular genetic breeding of high-quality, genetically improved rice. We report the positive regulatory factor *OsAAP6* for GPC to be significantly upregulated in *OsG6PGH1*(OX) transgenic positive plants, resulting in a significant increase in GPC and a significant decrease in starch content. This indicates that *OsG6PGH1* is involved in regulating accumulation of major storage substances in rice grains. Further observation of protein bodies in the endosperm of *OsG6PGH1*(OX) revealed no significant changes in the area and quantity of protein body I, and area of protein body II, but the quantity of protein body II increased significantly. This indicates that overexpression of *OsG6PGH1* significantly increases the number of protein bodies II, leading to a significant increase in GPC (**Figure 3**). Detection and analysis of expression levels of protein- and starch-metabolism-related genes in *OsG6PGH1* mutants and overexpressed transgenic endosperm (**Figure 4**) revealed expression levels of protein- and starch-synthesis metabolism-related genes in rice grains to be consistent with their corresponding main storage substances (protein and starch) contents. Proteomic analysis of *OsG6PGH1*(OX) endosperm revealed DEPs to mainly contain structural domains related to seed storage, have biological functions in metabolic processes, and participate in biological pathways such as carbon metabolism, and starch and sucrose metabolism (**Supplementary Figure 6**). Therefore, genes related to protein synthesis and metabolism are upregulated in *OsG6PGH1*(OX) transgenic positive endosperm, which can significantly increase numbers of protein bodies II, promote accumulation of related storage proteins in the endosperm, and increase GPC and nutritional quality.

Starch, an important component in rice grains, accounts for approximately 80%–90% of rice dry weight. This starch is composed of amylose and amylopectin (Zhao et al., 2022a), the composition and proportion of which directly affect grain quality. In rice endosperm, amylose is synthesized by the *Waxy* (*Wx*) gene encoding GBSSI (Zhang et al., 2019), and amylose content plays a decisive role in the eating quality of rice (Pang et al., 2016; Zhang et al., 2022). Inhibition of the branched starch synthesis related gene *SSIIa* leads to an increase in chalkiness characters, significantly affecting the appearance of rice (Zhang et al., 2011; Zhao et al., 2022b). The PPP is important for sugar metabolism in plants, and *G6PGH* is involved in biosynthesis and metabolism of plant starch. The cytoplasmic form of *G6PGH* is necessary for normal starch biosynthesis in maize endosperm. The *PGD3* mutation of the maize exhibits severe granule filling and defective embryonic phenotype (Ribeiro et al., 2020). We report the amylose content in rice grains of *OsG6PGH1* mutants to be significantly reduced, while the chalkiness rate, taste value, and gelatinization temperature all increase significantly. However, the amylose content of *OsG6PGH1*(OX) positive grains significantly increased, while the chalkiness rate, taste value, and head rice rate all decreased significantly (**Figure 5**). Therefore, *OsG6PGH1* can affect rice quality traits such as appearance, cooking, eating, and processing qualities by regulating contents of main storage substances (protein and starch).

*G6PGH* plays an important role in the response of plants to abiotic stress (Chen et al., 2020). Transgenic *G6PGH* in maize can significantly improve heat tolerance, thereby increasing maize yield under high temperature stress (Ribeiro et al., 2020). Overexpression of *6PGDH* in *Arabidopsis* significantly enhances cold tolerance (Tian et al., 2021). In rice seedlings treated with drought, cold, high salt, and abscisic acid, the transcription levels of *OsG6PGH1* and *OsG6PGH2* were upregulated (Hou et al., 2007). Under salt stress, expression of *OsG6PGH* in rice stems is upregulated (Huang et al., 2003), indicating that OsG6PGH actively participates in the response of rice to abiotic stress. We report *OsG6PGH1* to be neither localized on the cell membrane nor in the rice nucleus (**Figure 2**), but possibly in the cytoplasm, consistent with previous results. Analysis of expression patterns reveals *OsG6PGH1* to be a constitutive expression gene that is expressed in all rice tissues, consistent with expression of *OsG6PGH* in root, stem, and leaf tissues of cucumber (Wei et al., 2010). We report salt resistance in rice seedlings with *OsG6PGH1* homozygous mutants to be significantly reduced, but for salt resistance in *OsG6PGH1*(OX) positive seedlings to be significantly enhanced (**Figure 6**), consistent with expression levels in corresponding salt stress-related genes. Therefore, under salt stress conditions, *OsG6PGH1* can positively regulate expression of genes related to the salt stress response, thereby improving rice salt stress resistance.

In summary, *OsG6PGH1* was screened, identified, and validated to interact with *OsAAP6* through yeast one-hybrid technology, and *in vivo* point-to-point rotation and local surface plasmon resonance tests. *OsG6PGH1* is a constitutive expressed gene, and its encoded *OsG6PGH1* may be located in the cytoplasm. Expression levels of *OsAAP6* were significantly upregulated in *OsG6PGH1*(OX) transgenic positive plants, resulting in a significant increase in grain protein content (GPC), and improved nutritional quality of rice. *OsG6PGH1* positively regulates amylose content, and negatively regulates rice chalkiness rates and taste values, significantly affecting various quality traits such as appearance, cooking, and eating qualities of rice. Additionally, *OsG6PGH1* is involved in regulating the expression of salt-stress-related genes, enhancing the salt-stress tolerance of rice. Therefore, *OsG6PGH1* affects multiple quality traits, participates in the salt stress response of rice, and provides important genetic resources for molecular design and breeding of high-quality and multi-resistant rice varieties.

## Data availability statement

The original contributions presented in the study are included in the article/**Supplementary Material**. Further inquiries can be directed to the corresponding authors.

## Author contributions

BP: Writing – original draft, Writing – review & editing, Data curation, Funding acquisition, Investigation, Methodology, Project administration. YL: Conceptualization, Data curation, Formal analysis, Methodology, Writing – original draft. JQ: Data curation, Formal analysis, Methodology, Writing – original draft. JP: Conceptualization, Data curation, Formal analysis, Visualization, Writing – original draft. XS: Conceptualization, Data curation, Methodology, Writing – original draft. XT: Data curation, Methodology, Resources, Writing – original draft. ZZ: Formal analysis, Methodology, Resources, Writing – original draft. YH: Funding acquisition, Investigation, Methodology, Writing – original draft. RP: Formal analysis, Investigation, Supervision, Writing – original draft. WZ: Conceptualization, Data curation, Methodology, Writing – original draft. JZ: Data curation, Formal analysis, Investigation, Methodology, Writing – original draft. YS: Data curation, Formal analysis, Investigation, Writing – review & editing. QW: Data curation, Investigation, Writing – review & editing.
