# Supplementary material for: OsG6PGH1 affects various grain quality traits and participates in the salt stress response of rice
Source: Front Plant Sci. 2024 Jul 10;15:1436998. doi: 10.3389/fpls.2024.1436998 (PMC11267625; doi:10.3389/fpls.2024.1436998)
Supplement: Supplementary file 1 [file DataSheet_1.docx]

**Table S1.** Primer sequences used for subcellular localization

| Primer name | Primer sequence（5'-3'） |
| --- | --- |
| *OsG6PGH1* | F: CGCGGATCCAGATCTCATCTTACGCAGGC  R: CGGGGTACCTACAAGCGACTCAGCAGCAA |

**Table S2.** Primer sequences used for protein related genes

| Name | Primer sequence(5'to3') |
| --- | --- |
| *10KD Prolamin-F* | TGCAGTATTTCCCACCAACA |
| *10KD Prolamin-R* | ACATGAACATGGCTGTGGAG |
| *GluB1-F* | GCCAAAGTCAGAGCCAAAAG |
| *GluB1-R* | GAACCAATGTGCAACACCAG |
| *GluA1-F* | CATTTGAGCCAATTCGGAGT |
| *GluA1-R* | GGCCTGATTGTTGGAACTGT |
| *11S Globulin-F* | CACCAAACCCGATCTTCAGT |
| *11S Globulin-R* | CGGAACAGCTTCTCCATCTC |
| *Glutelin4-F* | GTCTAGACGAGATTATGT |
| *Glutelin4-R* | CCTTGTGATCCTTCCAC |
| *AlaAT-F* | CCATTTCTCGAGCAACAACA |
| *AlaAT-R* | ATTTGCAGGGTATCCGTCAC |
| *GluA3-F* | TGAAAACCAACCCTGACTCC |
| *GluA3-R* | ACTCATCTCCCCTCTTGTGC |
| *GluA2-F* | GCAAGAGCAGGAACAAGGAC |
| *GluA2-R* | CCTCATGGTGCAAAAGGTCT |
| *GluB4-F* | GCGACCAGAAGGCTACAAAG |
| *GluB4-R* | TTGCTTGTTGATCGTTGCTC |
| *RA16-F* | AGGTAGTGATCTCGGCGTTG |
| *RA16-R* | CCGATTCCTGGCTGACATAG |
| *β-actin-F* | TGCTATGTACGTCGCCATCCAG |
| *β-actin-R* | AATGAGTAACCACGCTCCGTCA |

**Table S3.** Primer sequences used for starch related genes

| Gene | Primer sequence(5'to3') |
| --- | --- |
| *SSI-F* | TCATGGATGTGAAGGAGCAA |
| *SSI-R* | TGGCAGTGAACCACAAACAT |
| *Susy3-F* | CATGTACCCCCTGCTCAACT |
| *Susy3-R* | GTCAGCTGTAATGCCTGCAA |
| *SSIVa-F* | GGGAGCGGCTCAAACATAAA |
| *SSIVa-R* | CCGTGCACTGACTGCAAAAT |
| *Susy2-F* | GCTGAAGGACAGGAACAAGC |
| *Susy2-R* | CACCACAGACAACCACAAGG |
| *SBE-F* | GGCATTGCACTCCAAAAGAT |
| *SBE-R* | GCTCCAGTTGTTGCCTTCTC |
| *AMY3A-F* | CCCAGGAGTACCATGCATCT |
| *AMY3A* -*R*  *AMY3B -F* | CTTGGTGATGACCCTCTCGT AGCGGTCTCAGAGTTCCTGCA |
| *AMY3B-R* | TCAAATCTTATTCCAGGCACCA |
| *ISA1-F* | TGCTCAGCTACTCCTCCATCATC |
| *ISA1-R* | AGGACCGCACAACTTCAACATA |
| *ISA2-F* | TAGAGGTCCTCTTGGAGG |
| *ISA2-R* | AATCAGCTTCTGAGTCACCG |
| *GBSSI-F* | TTGCAGACAGGTACGAGAGG |
| *GBSSI-R* | TCAACTCCAGTGTCAGGTCC |
| *β-actin-F* | TGCTATGTACGTCGCCATCCAG |
| *β-actin-R* | AATGAGTAACCACGCTCCGTCA |

**Table S4.** Quantitative primer of salt stress related genes

| Name | Primer sequence(5'to3') |
| --- | --- |
| *OsPEX11-F* | GCGTCTACTACTTCCTCG |
| *OsPEX11-R* | GACTCCAGTTTGCCGATC |
| *OsJRL-F* | AGGCGTGACAATCTACAG |
| *OsJRL-R* | GGTTCCAGAAATCTCCTTGA |
| *OsHKT1-F* | ACACCCAATATTATTCCTCTTAA |
| *OsHKT1-R* | CGGGAATACGCTAAAGG |
| *OsAKT1-F* | AGAGATCCTTGATTCACTGCC |
| *OsAKT1-R* | TCTACTAACTCCACACTACCAG |
| *Salt-F* | CGAAATAATGTTCCATGGTGTT |
| *Salt-R* | TGTACTACGGATCGGTGCAA |
| *OsWsil8-F* | TGTGACTCGATCCAGCGTAG |
| *OsWsil8-R* | GTTCCTGCTGAGAAGCCATC |
| *β-actin-F* | TGCTATGTACGTCGCCATCCAG |
| *β-actin-R* | AATGAGTAACCACGCTCCGTCA |

**Table S5.** Differentially expressed proteins associated with starch and protein metabolism

|  | Protein ID | Gene name | Gene ID | Function | Protein regulated |
| --- | --- | --- | --- | --- | --- |
| 1 | Q43009 | *OsSUS3* | LOC_Os07g42490 | Carbon distribution within the grain filling | down |
| 2 | Q688J4 | *SBDCP2* | LOC_Os05g37450 | Involved in starch biosynthesis | down |
| 3 | Q7G065 | *OsAPL2* | LOC_Os01g44220 | Involved in starch biosynthesis | down |
| 4 | Q6AVA8 | *OsPPDKB* | LOC_Os05g33570 | Involved in endosperm development | down |
| 5 | Q93X08 | *OsUgp1* | LOC_Os09g38030 | Involved in carbohydrate metabolism | down |
| 6 | Q10LP5 | *OsSUS4* | LOC_Os03g22120 | Regulation of starch metabolism by sucrose | down |
| 7 | Q9AUQ4 | *OsPGM* | LOC_Os03g50480 | Involved in the breakdown and synthesis of glucose | down |
| 8 | Q0DDE3 | *SSIIa* | LOC_Os06g12450 | Involved in endosperm starch synthesis | down |
| 9 | Q0DC10 | *OsGWD1* | LOC_Os06g30310 | Involved in leaf starch degradation | down |
| 10 | B7FA07 | *OsEno1* | LOC_Os09g20820 | Involved in carbohydrate degradation | down |
| 11 | Q01401 | *SBE1* | LOC_Os06g51084 | Involved in starch anabolism | down |
| 12 | Q01881 | *RA5* | LOC_Os07g11510 | Seed storage protein | up |
| 13 | Q9FXT8 | *OsRPT4* | LOC_Os02g10640 | Involved in protein synthesis hydrolysis | up |
| 14 | Q7XCL2 | *RIF2* | LOC_Os10g39620 | Encodes protein | up |

There are 14 functionally characterized rice genes ([https://venyao.xyz/funRiceGenes/](https://links.email.frontiersin.org/ls/click?upn=u001.qPvnClRwyVsaBj5SNX-2FU6r7qvChw91pGO6IUSeDoloYV-2FPyjsWsLHBoBEsW-2B1HdzO5Xp_YD2OjO1OG-2BDC8gf8vZ-2B-2F5Nz5l392svjPaa7RvfyyBVw2MposwiXi3PZaTtrV2xzSuLkKyGwc76IJkSkI3FKmD-2Fr0vCuVMNEwEnSXELElJNleGc4yTL3pxGAbbYT-2FMcTsCxCIsUwMiMtieNUmQNqJO8-2BNzvJcybn3mc94iZN8FLZGl-2BlISybhADIClXCwobpSuPLX-2BoN2g1jS00zWyyHYsZrahHFbLuam0GLbdbIjzEPkKz-2F-2B4rhFZyuF8vC0rEStBLqVcuD-2FS0tl4vfAPWomgEZgjYmd7SB6lSCgBbz2Z2A45P9b-2F2bLkWo4ImN34opZ8BSTPPfAu0NxpqpRVdACZer1LSQ3mNPqCX2vYVC3Bni1QqcapOLcmiqm6vQaBs85YZRY5K-2F70mX6UNQd02j5YQ-3D-3D)) in the differentially expressed proteins (involved in grain protein and starch metabolism processes) identified.


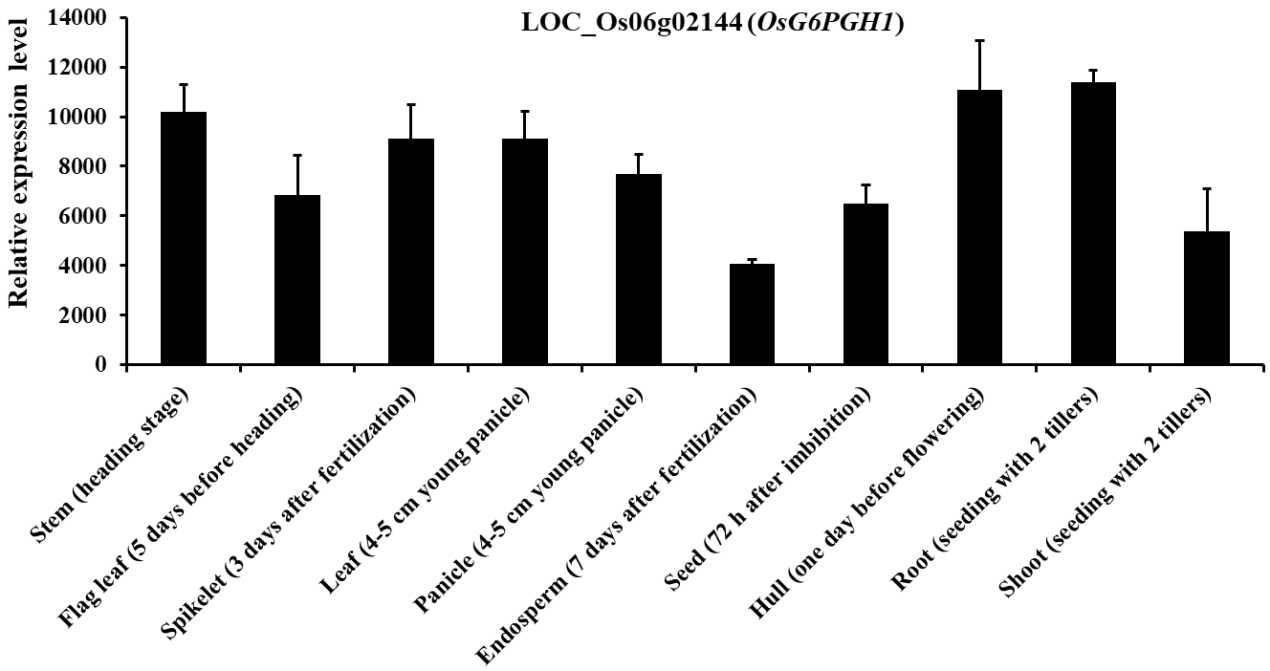


**Fig. S1.** The distinct tissue expressions of *OsG6PGH1* (LOC_Os06g02144) on CREP (http://crep.ncpgr. cn/crep-cgi/query_by_tree.cgi), and this result is in accordance with Fig 2A.


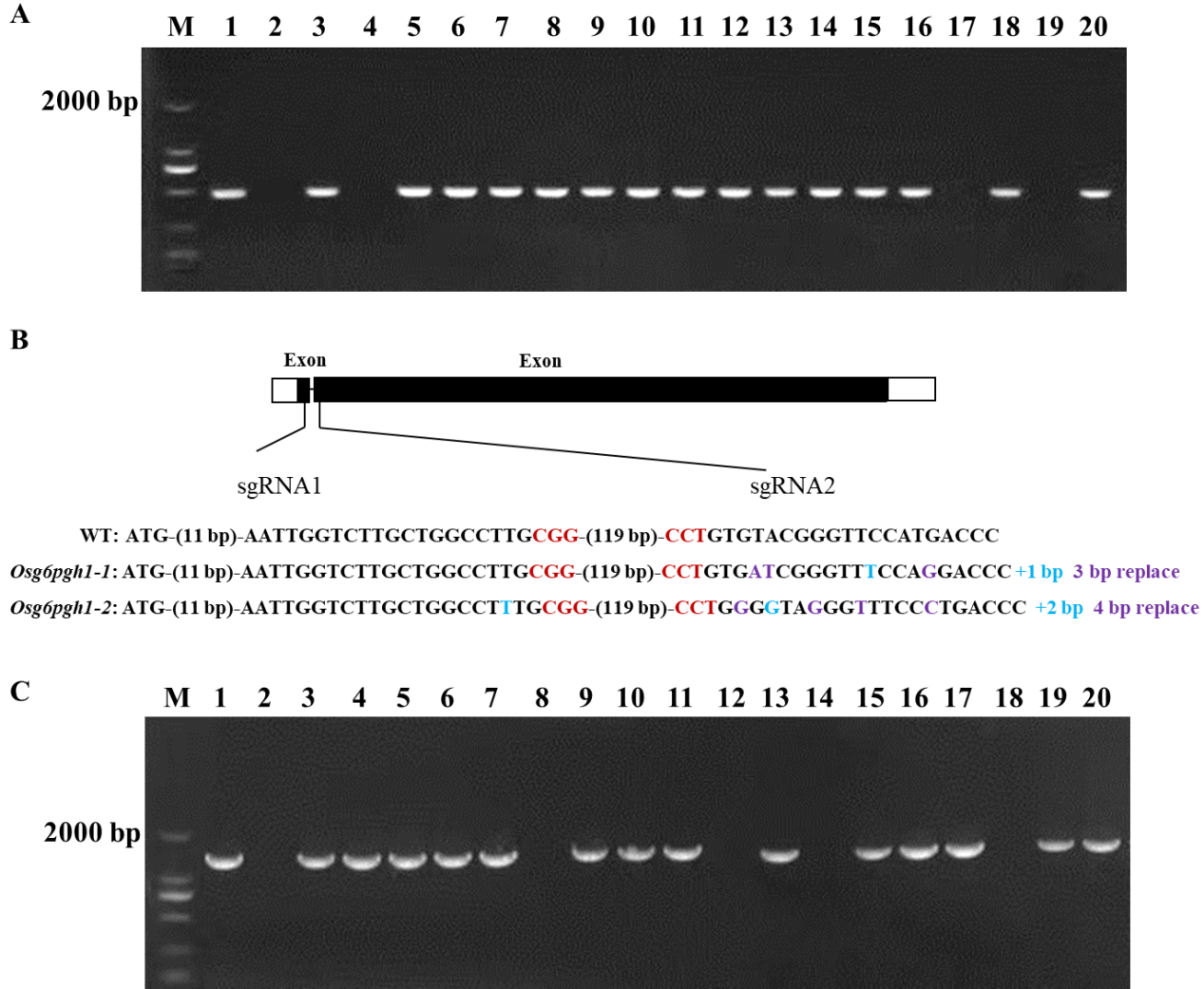


**Fig. S2.** Creation and detection analysis of *OsG6PGH1* transgenic rice lines; (A) positive identification of mutants; (B) gene-edited plant sequencing analysis: black boxes(exons), and lines (introns); white boxes represent non coding regions. (C) Positive identification of transgenic plants of overexpressing *OsG6PGH1*. M represents DNA Marker, 2000 bp; 1–20 represent swimlanes.


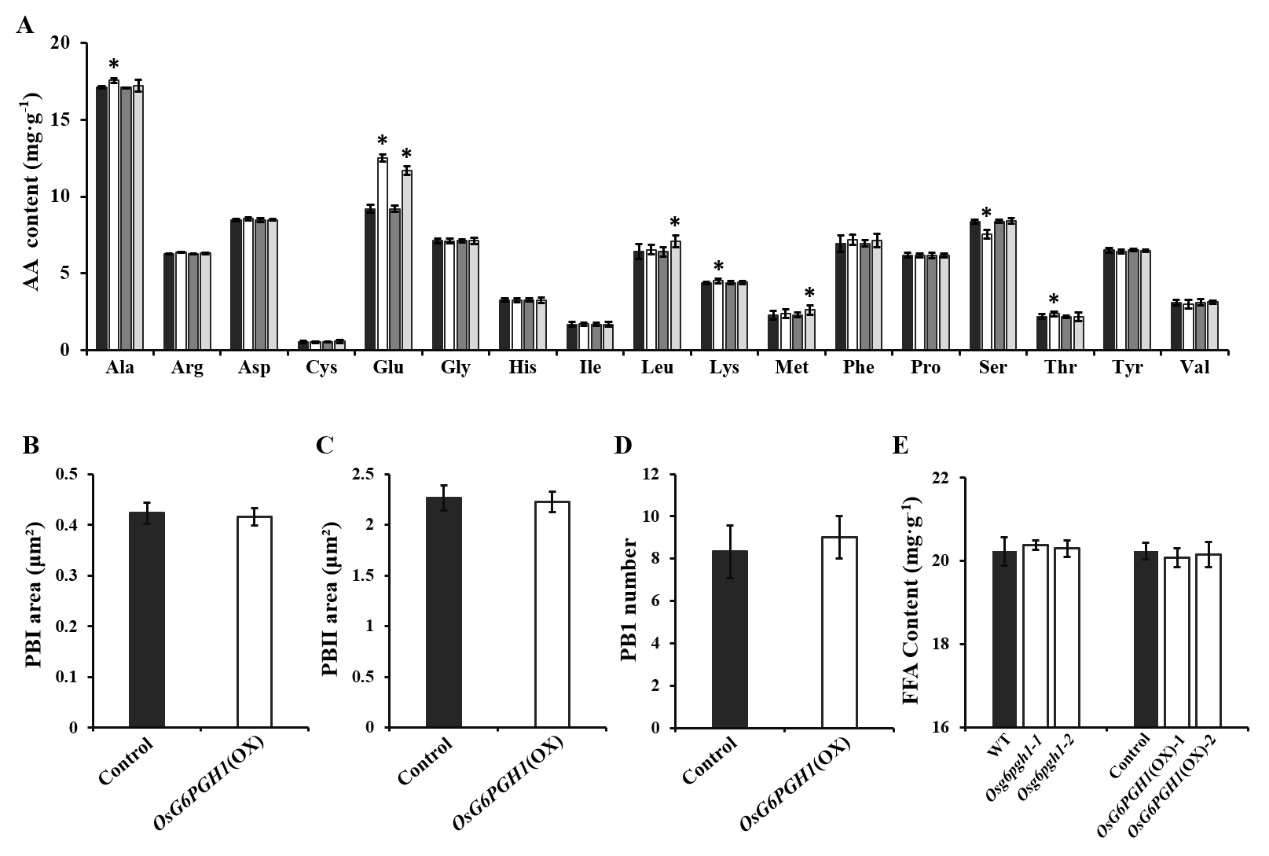


**Fig. S3.** Detection and analysis of amino and free fatty acid contents, and protein bodies in grains of *OsG6PGH1* transgenic plants. (A) Detection and analysis of amino acid contents. Average cross-sectional area of (B) protein body I, and (C) protein body II; (D) quantities of protein body I. (E) Detection and analysis of free fatty acid contents. Significant differences are based on two-tailed *t*-tests, where ***P* ≤ 0.01, **P* ≤ 0.05. WT, Wild type; Control, *OsG6PGH1* overexpression transgenic negative control. Error bars, standard error of the mean (SEM).


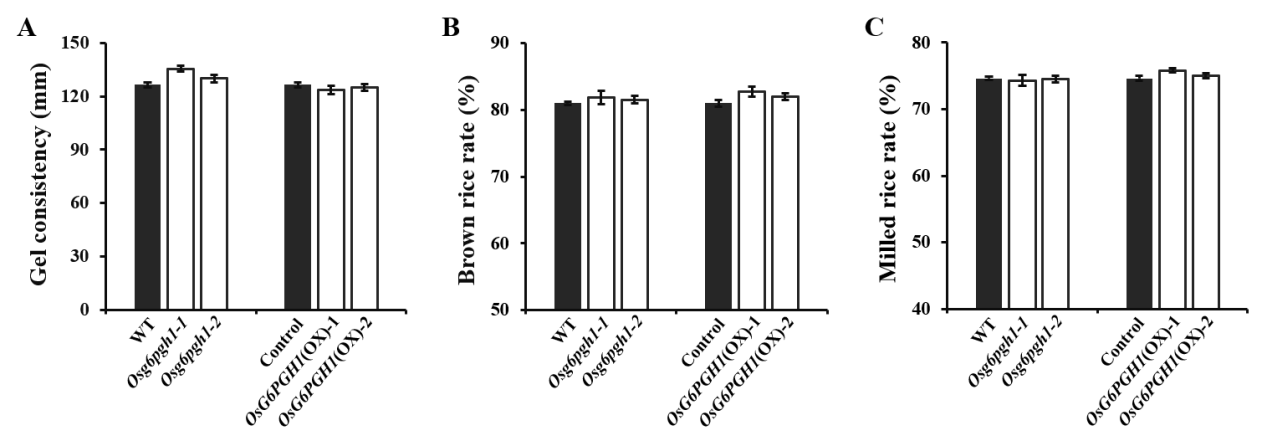


**Fig. S4.** Analysis of gel consistency and partial processing quality traits of *OsG6PGH1* transgenic grains. Detection and analysis of (A) rice gel consistency, (B) brown rice rate, and (C) milled rice rate. Significant differences are based on two-tailed *t*-tests. WT, Wild type; Control, *OsG6PGH1* overexpression transgenic negative control. Error bars, standard error of the mean (SEM).


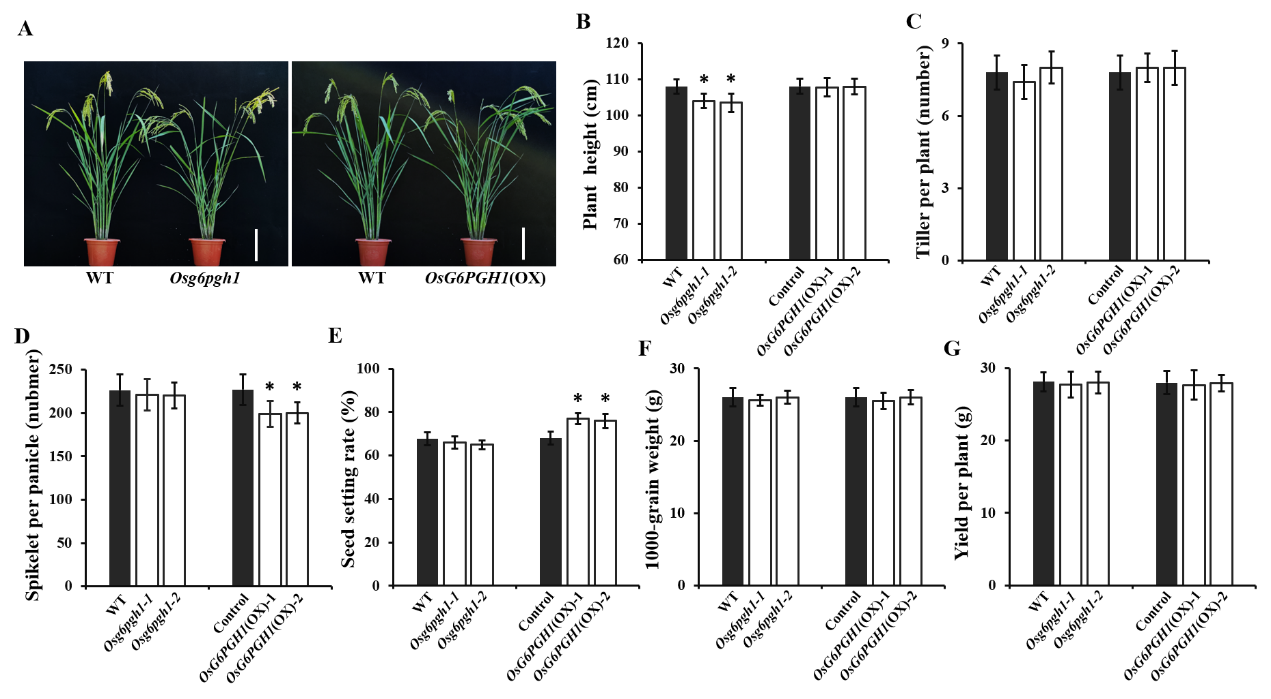


**Fig. S5.** Analysis of agronomic traits of *OsG6PGH1* transgenic plants. Plant: (A) phenotype, (B) height detection and analysis, (C) tiller number per plant, (D) spikelet number per panicle, (E) seed setting rate, (F) thousand grain weight, and (G) yield per plant. Significant differences are based on two-tailed *t*-tests, where **P* ≤ 0.05. WT, Wild type; Control, *OsG6PGH1* overexpression transgenic negative control. Error bars, standard error of the mean (SEM).


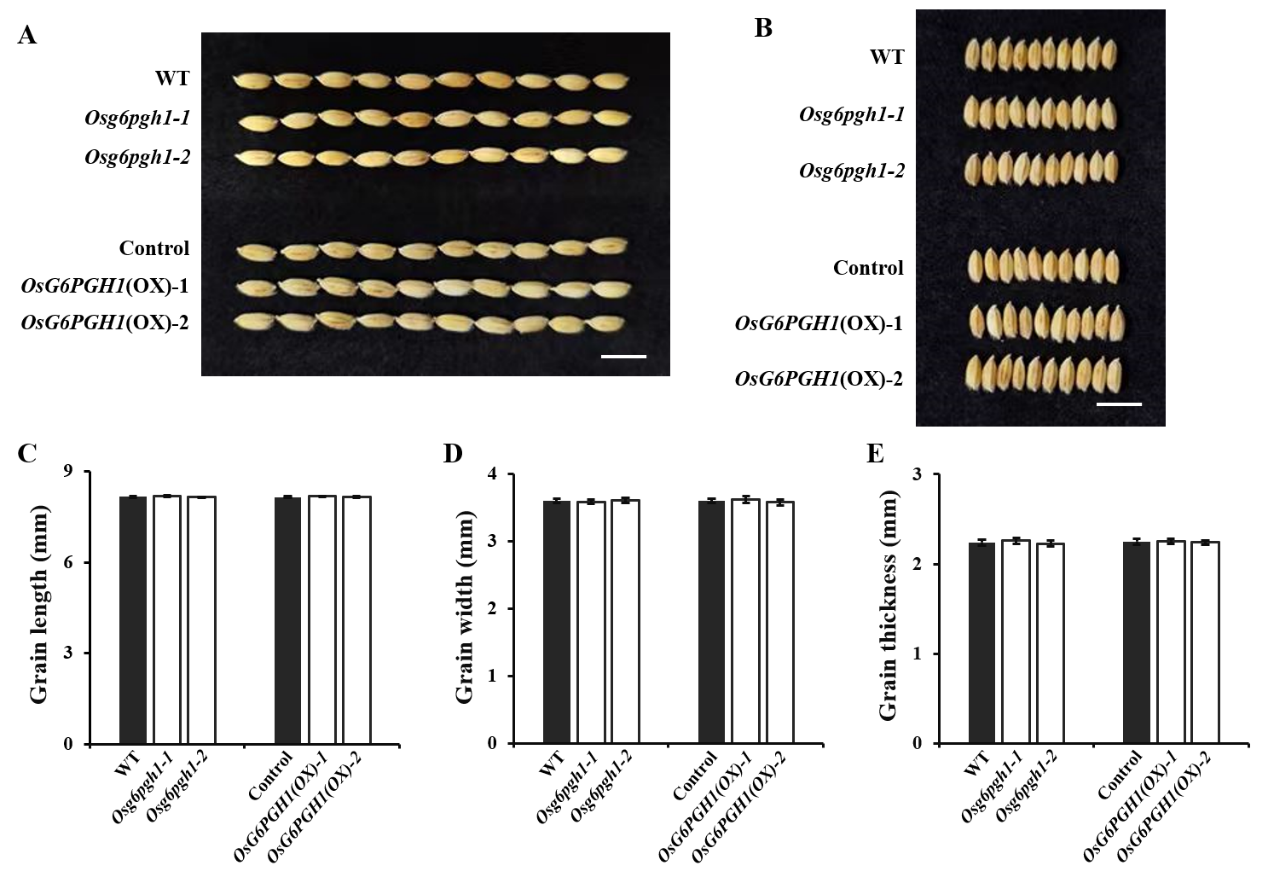


**Fig. S6.** Detection and analysis of grain types of *OsG6PGH1* transgenic seeds. Phenotype: (A) grain length, (B) grain width. Analysis of grain (C) length, (D) width, and (E) thickness. Scale bars, 1 cm. Significant differences are based on two-tailed *t*-tests. WT, Wild type; Control, *OsG6PGH1* overexpression transgenic negative control. Error bars, standard error of the mean (SEM).


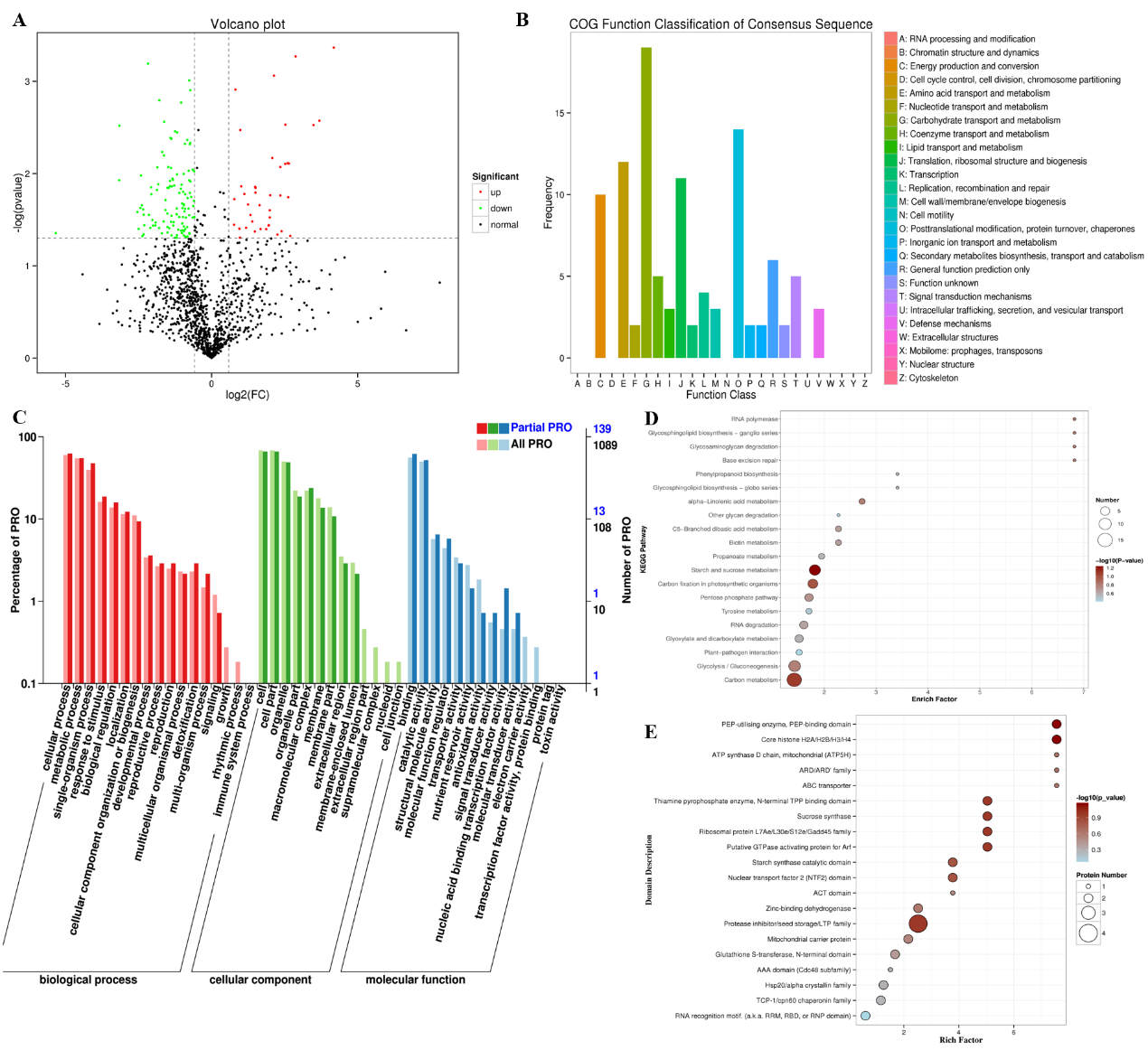


**Fig. S7.** Proteomic analysis of *OsG6PGH1(OX)* endosperms. (A) Volcano plot of differentially expressed proteins (DEPs), with each point representing a gene. (B) COG functional annotation analysis, (C) GO functional enrichment analysis; and DEP enrichment analysis of (D) KEGG pathway, and (E) Pfam domain.
